# Fogyism in Physic

**Published:** 1865-02

**Authors:** W. C. Munson


### FOGYISM IN PHYSIC.

**An Address delivered before the Cairo Medical Society, and published in compliance of a vote of the Society. By the President,**

W. C. MUNSON, M. D.

The preservation of health, the prevention and removal of disease, are perhaps the most interesting and absorbing subjects which can claim our attention. Hence great and philanthropic minds, from Hippocrates down to the present time, have (by laborious research and experiments) built up what is called a "medical profession." Candor, however, compels the admission, that a spirit of bigoted *exclusiveism* has been, and is yet too frequently found among its members.

When success of *party* is more coveted than the triumph of truth, this spirit is fostered; and free and liberal investigation is stifled. From this exclusiveness we wish to be free. In this age of progress, when the foundations of political, and even ecclesiastical superstructures are so closely scanned, the members of this noble profession should put their seal of disapprobation upon all the *despotic edicts* of "Medical Associations," either *National* or *sectional*, which declare that every physician shall be strictly *regular* in his practice, and follow the course laid down by his *master*, else himself and students shall be proscribed from the profession, and excluded from Medical schools, where such free-thinking *heretics* might corrupt the young and unsophisticated *lambs* of the *flock*. Believing such rules better adapted to the government of infants than men, we would say—let them be *abolished*.

Freedom is needed to think. Liberty is demanded—liberty to investigate calmly, without fear or favor, and reject all old and crumbling errors, though called *science;* whether upheld by *Authority* or not. Liberty to travel out into the unexplored fields of nature and gather such instruction as she may place within our reach. When "Medical men" shall discard the appellation "Allopathy," or any other name indicative in the popular mind, of a *party* in the profession, our ears will not be offended by the question (so grating upon the auditory nerves of the man of science,) "What system do you practice?" Why there is but one system of practice. Among scientific men there necessarily can be but one, which is, of course, the "*Curative System*," and consists in the employment of the most efficient therapeutics within their reach. As a matter of course, the kind of therapeutical means employed will depend very much upon the diagnosis. Everything available within his reach may be "pressed into service," and made to "do duty" for the physician who is more devoted to the development of truth, than the advancement of *party*. To *him* there is nothing interdicted.

Doubtless the parties which have originated in the profession, and the exclusiveness which party spirit engenders, has bound many active minds in a kind of mental *incubus*, from which they never escape, and but for which, might have added largely to the resources of the profession. At the present day, when such a vast amount of published matter must be *sifted*, in order that the greatest amount of truth may be elicited, we need freedom to look around, compare, analyze and investigate, that a full array of facts may be *marshaled*, to aid in the adoption of truth, and the rejection of error and *quackery*.

It could hardly be expected, however, that the student, in the commencement of his explorations in the vast field of medical science, should be competent to make judicious selections of *text books*; and will save much valuable time by having these selections made for him. The undisciplined mind is easily mislead, and the power of delusion is so great, its operations so extensive, and its evils so terrible, that every influence favorable to close investigation should be brought to bear. In short, it should be impressed, that *facts* not *theories*, should be trusted. The history of the world shows that the majority of mankind quietly yield themselves to every delusion which may be prevalent in the country where they were born. Tell me in what community a man was born, and I can tell almost to a certainty what principles he has adopted. In one community he will be a Roman Catholic, in another a Protestant, in another a Greek, in another a Jew, in another a Mahomedan, and so on, taking the hue of society, imbibing every delusion which surrounds him, as the tree imbibes the sap from the soil where it is planted. And these *opinions* or delusions, thus passively imbibed, are seldom received because supported by specious arguments, or tangible proofs, but too often by *influential* associations, conspiring against the people, or by the strong arm of military power.

At present, in Europe and America, various forms of Christianity are professed. But that we are called Christians—

that we are not repeating prayers with our faces towards Mecca—is probably only because, in the eighth century the Catholic armies were more powerful than the Mahomedans. Because Charles Martel, the mayor of France, in the great battle of the plains of Poictiers, in 732, succeeded in driving back the hordes of "Saracen invaders," whose armies at that time threatened to conquer all Europe. That man should be the passive creature of circumstances, believing what a king decrees to be true; that he should accept with all the fervor of his soul, whatever has been arranged for him to believe by kings and priests, is one of the melancholy and humiliating facts of history. The combined leaders of mankind in former times, and under the influence of monarchical governments, have looked upon the masses as mere puppets to be moved by the word of command. These sentiments and this policy still prevails in Europe; and has attached itself more or less to all that we have borrowed from Europe. We have borrowed from Europe much of the literature, rules, organization, sentiments and *esprit de corps*, of the profession.

So much of the above spirit as exists among us has probably been transplanted from the monarchical soil of Europe to the free soil of America. If it lives and flourishes here at all it is as an exotic, having nothing in common with the free spirit of our country. May it not be that the influence of Europe in this direction exists among us too much? Perhaps the idea is too much acted on, that the *leaders* must think for the masses, and that the *authorities* must be obeyed by their humble followers. The writer does not, by the above remarks intend disrespect for the *learned authorities*, or insinuate that their study is unnecessary, but simply that no fine-spun theories, however well they may appear on paper, or sound to the ear; that no weight of authority that may attach to great names, from Hippocrates and Galen, down to Benj. Rush, should induce us to receive their opinions without strict scrutiny, and unbiased and rigid investigation.

Perhaps teachers in Medical schools err, when they receive their young and impressible subjects from the country, and tell them that they must follow their great *authorities* and indorse all their doctrines. And does not the student (finding himself surrounded by the imposing influences of flourishing colleges and public sentiment, from which it is not easy to escape,) passively yield his soul to the guidance of his teachers, and become too much like clay in the potter's hands to be molded into shape? How seldom is heard expressed a manly sentiment in favor of free and independent investigation? More frequently is the idea of reverence for the *learned authorities* inculcated. And this, perhaps, becomes so impressed on some minds that it goes into the very structure of the soul. Like the image of himself, which Phidias cut so deeply into the buckle of his Minerva, that no one could obliterate it without breaking into fragments the statue itself. And thus through life they never undertake to investigate for themselves, the doctrines taught them in their youth. It is gratifying, however, to be able to state that *Fogyism*, as above portrayed, applies more to the past than the present; that in our Medical schools in America, at the present day, the above is the exception, not the rule.

Let there be no stone wall of *party* prejudice erected around us. Let it not be thought disreputable to take a careful survey of Homœopathy, Hydropathy, or Eclecticism, or give them credit for all the truth they may, individually or collectively, possess. In this day of progress and intelligence, we think it incumbent on every high-minded physician to refuse to be controlled by party restrictions or prejudices, and feel it a duty to thoroughly master and comprehend all the so-called *systems*, *parties* and *pathies*. But this would be *Eclecticism*, which has already appeared in the *arena*, claiming to be a distinct *party*. So far as the *name* is concerned, it is not objectionable, but commendable—what every accomplished physician is, eclectic. He culls and selects as his judgment dictates But when the term is applied to a party, as bigoted and exclusive as any other, and who insist that everything else in therapeutics must be superceded by some especial dogma of theirs, it is undoubtedly inappropriately applied.

We are inclined to deny "Papal infallibility" to any *party*, *pathy*, or *assumed system*, and when men of the most profound scientific attainments, in their most comprehensive and liberal investigations, have mastered all within their grasp, *they* will be in no danger of that shallow egotism which supposes all beyond to be folly and imposture, but will be willing to admit any truth that may be found in any or all *assumed systems*. Fogyism has, perhaps, been responsible for the origin of these *systems*, yet justice to all parties demands that "Young Physic" be held up for inspection, in order that we may see upon what ground it sets up its claim for exclusive favor. As such we may name Botanic, Hydropathic, and Homœopathic. Eclectic is not mentioned because, 1st. It is a *misnomer*, as the name can not be properly applied to a party of exclusivists, (though it is true that much bigotry and exclusivism assumes this name). 2d. Because the Botanic is its sire, and is, perhaps, sufficiently transparent to enable us to look through it, and see the features and physiognomy of its offspring, and recognize their consanguinity.

But to return to these systems, we have, 1st. A party of *chameleon* name, changing its hues and titles like the "dying dolphin," the Botanic. The absurdity of forcing therapeutics to follow in the wake of every visionary hypothesis, and "do duty" alike for the "solidist," the "humorist," the "Broussaist," and the empirical "routinist," (by a sort of natural reaction, perhaps,) brought out the ignorance, the common sense, and in some instances, useful medicines of Samuel Thompson. His limited pharmacopœia and universal application of steam, originated the name *Steamers*. It will, perhaps, be just to admit that cures were effected, some of which possibly may have been beyond the reach of remedies in the hands of other practitioners of that day. In an enlightened community the ignorance and limited resources of Thompsonianism could not maintain a hold on public confidence. But the Thompsonian or *Steam System*, in the hands of men of much more science than its founder, has progressed and improved far beyond the ideas of Thompson, and rising above the titles of Steamers and Thompsonians, has been designated as "Botanico-Medical."

Medical schools have also been acknowledged useful and necessary. Teaching, studying, and attempting to practice medicine, could not fail to have some effect upon them, and accordingly we find Botanico-Medical advancing still further beyond Thompsonianism, enlarging their resources and using many valuable remedies; but yet, making an outcry against some of the most efficient therapeutical means employed by the most eminent and successful practitioners, denouncing them as poisonous, and unfit to be retained in the officinal list. Perceiving that they could not rely on vegetable remedies exclusively, and that all minerals were not as bad as Thompson supposed, the title of Botanico-Medical was dropped and that of Physo-Medical substituted. This title, however, was rather awkwardly constructed, as *physo* signifies *air* or *wind*, and therefore, a *Physo-Medical* system must be rather too gaseous for solidity or dignity. After this defect had been discovered, Physo-Medical was laid on the shelf with Steam, Thompsonian, and Botanico-Medical, and *Physo-Pathic* is, I believe, taking its place, or has done so. But as this signifies a windy disease, or windy treatment, suggesting painful and colicky ideas, we "can't see" the improvement. Their bigoted exclusiveness and ignorance is seen in their idea of no-poison medicine. The non-poison platform is boasted of, and all are denounced who use "poison-medicine." If such a party would adhere to bread and water as their sole medicines, there would be consistency between their doctrine and practice. But when many of their drugs will kill even a well man in a short time, their theory becomes palpably absurd and inconsistent.

The non-poison theory would confine us to food and drink. By medicine we mean something not used as food, but designed to modify the vital functions, and there is no such substance in nature which is not poisonous, if used with sufficient freedom. The distinction between medicine and poison is a distinction of degree, not of kind. Any medicine, if sufficiently concentrated, would be called a poison, and any poison if sufficiently subdivided or diffused, possibly may act as a medicine.

Snake poison is converted into medicine by the Homœopaths, and the most harmless medicines would be pronounced poisonous, if concentrated until a single grain would be a fatal dose. The idea that a medicine is desirable because it can be given in a large dose, or unfit to be used because it must be given in a small dose, is certainly a very crude theory. If a medicine is found beneficial, use it; if not, reject it. If it be good, the smallness of the dose should be no objection; it is certainly more convenient to carry an ample supply in a small pocket vial than to "fetch in an armful of herbs and a kettle to boil them in." It is undoubtedly right to reject everything which acts harshly, and generally produces ill-effects, but the "Botanico-Physo-Eclectic-Medicals" have gone beyond these rational limits, and rejected articles which, in their legitimate use, are both safe and beneficial.

The Hydropathic or Water-cure system, also possesses some merit, and so far as it goes should be incorporated among the resources of every intelligent physician. But when water is made the sole agent in the treatment, and when a contempt for all other therapeutical means is expressed, and an unwarrantable prejudice is instilled into the public mind against all medicine whatever, we feel inclined to reject this form of delusion and prejudice. But if we must be narrow-minded, if we must dwell on one idea alone because there is not room enough in our minds for more, water is, perhaps, as good a hobby as any other. But why the necessity for intelligent men to thus surrender their general knowledge and resources? We might as well resolve to live on one article of food, as bread, or potatoes, or rice, or corn, or oats, as confine ourselves to one medicine. No doubt a Water-cure system would cure or relieve some cases, and so would a Lobelia-cure system, or a Podophyllin-cure system, or a Calomel-cure system, or a Steam-cure system, or a Brandy-and-salt-cure system. Thus every doctor might have his hobby, his own great cure-all. And there is no doubt that any single valuable drug in the whole *materia medica* would accomplish very much, if made a hobby, and pursued with energy and enthusiasm.

There is another hobby upon which respectable progress has been made, and which, like Hydropathy, has some merit, which is, mainly, that it has more fully demonstrated, than was ever done before, the power of nature, unaided, to overcome disease. It has also shown the enormous abuse of that *blind routine* practice, once so common. It may freely be admitted that Homœopathy, or more practically speaking, the use of infinitesimal doses, has the merit referred to. And if it was only added to the common stock of science, we would say to Homœopathy, as to Hydropathy, welcome! but unfortunately, these two worthy new-comers, after having been kicked out of the temple of Esculapius by the *old masters*, have got their spirits up, and each resolved that they will not only make their way into the temple, but will also reciprocate favors by kicking every body else out, and taking exclusive possession. Now against this, we protest, I should vote for the admission of Homœopathy and Hydropathy: Let them come in, and occupy as much ground as they can really cover. But we object to their turning anybody else out. When they undertake to reciprocate the arrogance of "Old Hunkerism" by the rival arrogance of "Young Hunkerism," they provoke our criticism. We are tempted to ask, What is this new system, which is to supercede everthing else, and fill the whole temple of Esculapius? It is only *similia similibus curanter.* But when it comes with the demand that we shall surrender at discretion—give up our arms upon which we rely—we beg leave to look at their documents, and see if they have a right to make any such demand. Practically, Homœopathy demands that you shall lay aside vigorous potential doses of drugs, and consent to use only little *globules* of sugar and milk, which have no medicine in them that can be detected by a chemist. Neither can it be detected in them by taste or smell ; but by a process of reasoning it may be determined that it ought to be there, because the sugar has been in the mortar in company with the physic, and might have got infected with the qualities of the medicine.

If a lump of sugar was held for one moment over a vial of cologne, or linseed oil, and then swallowed, more than a homœopathic dose would be taken.

Perhaps it should not be denied that Homœopathy has effected cures, (*i. e.*, Homœopathy according to their theory.) But how? can the *rationale* be explained? It may be answered, that the mind, fully admitting the curative power of the medicine, may send a stream of electricity or other force along the nerves, to the diseased organ, and the *vis medicatrix naturæ* effects the cure. We know that we can cure (or rather aid nature to do so) by doses that we can see, and feel, and taste, and which produce plain, intelligible and powerful effects, in accordance with common sense and sound philosophy, and therefore should not give up a sure reliance for the speculative beauties of Homœopathy.

Supposing that these infinitesimal globules and tinctures—tinctures, did I say?—about as strong as a teaspoonful of salt in the Ohio river; supposing that these essenses, shadows and ghosts of departed Acconite, Belladona and Mercury, had all the power claimed for them? it would amount to just nothing at all upon a healthy constitution. A lively baby would swallow the whole contents of the Homœopathic laboratory—a hundred pills of a hundred different kinds, making in all ten thousand doses—and perceive as little effect as if it were sugar candy.

This very delicate and wonderful method of overcoming disease by these spirits of departed medicines, which are quite invisible and imperceptible to the healthy, are supposed to become real "spirit rappers," when properly fitted to a disease; but how to fit them properly appears to be rather a delicate operation.

A learned Homœopathic author once declared that he would have cured one of his patients of an attack of pneumonia but for the fact that he had a hollow tooth in which the little sugar globule lodged, and consequently was unable to perform its great mission to cure the lungs. The wonderful powers ascribed to their doses is supposed to be developed by rubbing, by trituration in a mortar, or by shaking in a vial. How medicine is improved by shaking has never been explained. Should the patient be shaken, it might perhaps do him good sometimes. Hahnemann (who is almost worshiped by Homœopaths) declared that he rendered his medicines very powerful by shaking them; the longer he shook them, the more he stired up their wonderful powers, "until, by hard shaking, they became so furiously powerful as to endanger the lives of his patients," and he "was compelled to reduce the number of his shaking from ten shakes to two." It might, then, be asked, if a German dogmatist, who fancies because he has brought forward a new idea, (which, however, was originated long before his time; in fact, is found in the writings of Hippocrates,) shall be allowed by his mere arbitrary *dicta* to sweep away at one stroke everything accumulated by the therapeutic experience of ages, that his imaginary new principle may have sufficient room to display its beauty. It would be degrading to the profession to submit to such usurpation.

We would protest against cutting off any of our resources, water, potential concentrated drugs, infinitisimal medicated sugar, galvanism, animal magnetism, and all that can possibly be used with benefit. Whenever the physician resigns his freedom of choice, and abandons valuable resources, by joining a proscriptive party, he lowers his dignity as much as he diminishes his usefulness. Let us, then, proscribe nothing, reject nothing that can be made beneficial. Let physicians ignore all proscriptive parties, but retain all they collectively possess. Let Hahnemann stand on the merits of his own globules, and others likewise on the merits of their respective dogmas; and not boastfully exclaim, like Paracelsus, the "monarchy of physic is mine." There is no monarchy in medicine; there is no "master builder;" all are but "journeymen mechanics," working slowly; some, indeed, have excelled others, each one adding a little, some of good material and some of bad. We have been working some twenty odd centuries, and yet not half-built the temple of *Esculapius*. He who would claim to have built the temple, is an imposter. The true physician, then, can become neither Pressuitzians, Hahnemanians, Thompsonians, Allopaths, or Eclectics; but intelligent medical gentlemen, who think for themselves, and "call no man master."

